# Supplementary material for: The validity and reliability of school-based fundamental movement skills screening to identify children with motor difficulties
Source: PLoS One. 2024 Feb 15;19(2):e0297412. doi: 10.1371/journal.pone.0297412 (PMC10868745; doi:10.1371/journal.pone.0297412)
Supplement: Attachment — Submitted filename: Response to Reviewers.docx [file pone.0297412.s001.docx]

Editor Comments

1. Please ensure that your manuscript meets PLOS ONE's style requirements, including those for file naming.

We have updated the manuscript to reflect PLOS ONE formatting

2. Remove funding information from acknowledgements. Include amended funding statement within cover letter. Specify where the minimal dataset is held in the cover letter

This has now been removed from the acknowledgements section and both of these things are now included in the cover letter.

You should provide more details on sampling procedure and criteria for selection
This information has now been clarified in the methods section (see pages 5 and 6, lines 109 - 125).

3. I have to agree with Reviewer number 1 that your hypotheses at the end of the Introduction section seem identical to the results you present in your work.
   These hypotheses were not pre-registered so have been removed.
4. Also, authors should reevaluate appropriateness of statistical tests used in the study as indicated by both reviewers. Furthermore, we recommend the authors to revise terminology used throughout the manuscript

We have taken on board Reviewer one’s comments and kept in the classification analyses, but removed intraclass correlations (replaced with Kappa for inter-rater reliability) and logistic regression from the manuscript.

5. At the end of your revision, please, tailor study limitations accordingly

The discussion has been modified to include limitations highlighted by the reviewers (see pages 16 & 17 , lines 332-335, 345-354).

Reviewer 1

*General comments*

1. The authors often report statements that are not substantiated by the references they provide. I would encourage the authors to not report personal opinions and focus on the research question assessed in the article.

We have reframed the introduction and discussion to ensure that the findings of the papers cited are reported with more clarity, and included further references to support statements made (see pages 3-5 and 14-18).

2. Do not use unnecessary acronyms such as FMS, EACD, COSMIN, P.E., SEND. Please spell these acronyms out throughout the manuscript to improve readability.

All acronyms (barring the Movement Assessment Battery for Children, as this is a particularly well known acronym within this research area) have now been removed from the manuscript.

3. Details about the sampling, inclusion and exclusion criteria are insufficient.

I believe the choice of the statistical methods not to be appropriate, and lacking a sound rationale for the purpose of validating a screening tool.

Inclusion and exclusion criteria have now been clarified, and a flow diagram for sampling has now been included (as mentioned in a later comment). See pages 5&6 116 - 125. These analyses were chosen based on their frequency of use within the field for similar research questions. We appreciate your comments around more appropriate analyses, and have consequently removed the intraclass correlation results and logistic regression results from this paper. Instead, we focused on classification analyses and kappa statistics, as suggested in your comments below.

*Abstract*

1. P2, lines 22 & 27. Since this is a validation study, the term “Explore” should be replaced with “Assess”.
   Thank you for this helpful clarification of the most appropriate terminology - this has now been changed throughout the manuscript.
2. P2, line 35-36. Please remove this sentence “Universal screening of motor skills could help to reduce healthcare inequalities and improve life chances for children” from the end of the abstract. This statement is not substantiated from your data and is outside the scope of this validation study.
   On reflection, we agree that this is beyond the scope of the paper and this has now been removed.

*Introduction*

1. P3, line 45. Please provide a reference for this statement “can ultimately impact social mobility.”

References have now been included in support of this statement (see page 3 line 49).

2. P3, lines 55-56. Please remove this specification “(as if another disorder decreases the impact of motor deficits)” unless the authors can point the readers towards a critical perspective article on this issue.
   This has now been removed.
3. P3, lines 56-58. Please provide a reference for the statement “All of these factors combined mean that children with motor difficulties are not having their needs identified (most prominently children of ethnic minorities from deprived areas)”
   This paragraph has been revised in alignment with the comment below.
4. The first part of the introduction is generally too long. While it is important to stress the importance of the screening tool, I think this part can be shortened without impacting the focus of the work and without harming readability

We agree there was room to be more succinct in this paragraph, whilst still maintaining key messages. We have now edited this section to reflect this request (see pages 3-4).

5. P4, lines 75-76. Please provide a reference for “FUNMOVES allows two members of teaching staff to 76 assess the FMS of a whole class (~30 pupils) in under an hour.”
   Apologies that it was not clear - the reference in the next sentence was to cover both. I have referenced this paper twice now for clarification.
6. Was the study pre-registered? The hypotheses at the end of the introduction seem very specific and tailored to the results obtained. Either the authors provide a link to a pre-registration or I think this section does not help in reading the article.
   These hypotheses were not pre-registered, so have been removed.

*Methods*

1. P5, lines 103-104. The authors report that the first school to respond favourably was included in this study. It is unclear what happened to the other schools. Is other data available from the other schools?

There are no other schools involved in this study. This was a function of the timescale within which the study had to be run, due to funding limitations. This section has been reworded for clarification. See page 5 lines 109-115.

2. P6, lines 112-114. Please provide more details on how the sample size needed was estimated

The authors have reviewed this and feel that sufficient details have been included in this section. We are happy to be guided by the reviewer if they believe further information is necessary.

3. P8, line 176. Specify which version of R and RStudio was used for the analysis.

This has now been included (see page 9 line 199).

4. The authors use ICC to measure the agreement between MABC-2 and FUNMOVES, and between teachers and researchers. I do not understand the rationale for this choice. The MABC-2 has been defined by the authors as the gold standard, and a comparison between a test and a gold standard should be based on Accuracy, Sensitivity, and Specificity. On the other hand, agreement between raters is generally evaluated using Kappa statistics. It is also possible to assign weights to different types of errors. If instead the issue is that the tests provide numeric results, the assessment should focus on metrics such as RMSE or graphical visualization through a scatterplot. Please either consider more appropriate metrics or provide a sound rationale for your choice.

We appreciate your comments and your suggestions for more appropriate analyses. In response we have modified the analysis in line with your recommendations (see response to general comment 3), see page 9, lines 201-220.

5. P9, lines 184-185. “Logistic regression was used to establish whether FUNMOVES and the MABC-2 identified the same children as having poor FMS.” Why is this needed? Was the logistic regression used to adjust for some variables? See the point above.

We thank the reviewer for highlighting the redundancy of this analysis. We have now removed the logistic regression analysis to improve clarity and readability.

6. I understand that the use of percentiles by year group to define individuals with a condition is customary in your field. But several studies have shown issues with standardization and correction in other fields (eg. measurement of lung functions, measurement of hearing functions, measurement of cognitive decline). Please be aware of these issues.

We thank the reviewer for bringing this to our attention. We have now included this as a discussion point (see page 17, lines 345-354).

7. P9, Table 1. The metrics described in Table 1 are known to the broad audience. Please, remove the table and describe in the text the cut-offs used.
   These are not regularly used metrics within our field, and thus, we deem it necessary to include this table within the paper.

Results

1. Please provide a flow-chart indicating the number of contacted students and the number of students excluded by different reasons at different stages of the sampling procedure. This is a general recommendation for all observational studies (eg. STROBE guidelines). Information about how the authors arrived at the final sample is insufficient and does not allow judgements about representativeness.

There were no exclusions in the data. No non-consents were returned to the school so all children that were present on the days of testing took part. This has now been clarified (see page 10, lines 225-235). As there were no exclusions a flow chart was deemed excessive. It would only illustrate the number of children on the school roll, as all were invited to participate, flowing to a second box showing the number of children who did participate.

2. P10, line 202-204. This sentence belongs to the methods section or the discussion.
   We agree this gives context to the demographic of the school, rather than it being a result. This has now been moved to the methods section. See lines 97-99.
3. P10, Table 2. Please be consistent in your terminology: the term “sex” was used in the manuscript.
   Apologies for the oversight, Table 2 has now been amended.
4. P10, Table 2. report the number of individuals by sex along with the percentage (not the ratio).

We thank the reviewer for recognising the difficulties that may occur when interpreting this table - we have now included n and percentage (where appropriate) - see page 11.

5. The MABC-2 was only assessed for students judged as having motor difficulties by their teachers. Therefore, the assessment of accuracy has limited value. This should be emphasized in the Discussion.

We appreciate the limitations with this methodology, however it was necessary due to time and resource limitations. We’d also note that the sample referred by teachers also included children that did not have motor skill difficulties (aligning with literature showing that teachers have a poor understanding of motor development). We have acknowledged this in the discussion (see page 16, lines 332-335).

6. Please, report confidence intervals for all the reported metrics (Sensitivity, Specificity, PPV, NPV, Accuracy). The sample size is very low and this should be reflected in a measure of variability. This assessment should, in my opinion, be the main point of the paper together with a Kappa Statistics about the raters agreement (see points above).
   We agree that a measure of variability should be included, we have reported 95% confidence intervals in Table 5 (page 14).

*Discussion*

1. P13, line 247. This is not a “psychometric” test
   Correlation is not a measure of agreement. It measures linear relationships.
   Correlational analyses have now been removed from the paper and this wording has been amended (see page 14, lines 285-287).
2. P15, line 291-296. The use of norms by demographic characteristics has been shown to be problematic (see point above) and I do not think that your work “show that such programme of work is well justified”.

We appreciate the reviewer's opinion. However positive results in a preliminary study such as those presented in this manuscript (where the limitations of this are made explicit) do justify further exploration, in larger sample sizes. Regardless of whether percentile ranks are appropriate, we would argue that having more data (i.e. information on ability levels) will only increase sensitivity when comparing ability levels. We have included the limitations of using percentile ranks in the discussion (see page 17 lines 345-354).

3. P16, line 324-326. The final sentence in the conclusion is completely outside the scope of the article and should be removed.
   This has now been removed.

Reviewer 2

1. A database needs to be attached.

Information on where to access the database has now been given to PLOS ONE for inclusion in the manuscript.

2. There is no total score and description for FUNMOVES and MABC-2

This information has now been included on page 9, lines 217-219.

3. It is unclear how logistic regression was used? What is the outcome in logistic regression.

This analysis has since been removed, based on the recommendation of Reviewer 1.

4. State the applied statistical tests in the methodology
   This information can be found on page 9, lines 199 - 220.
5. It is not adequate to report the mean and SD when the coefficient of variation is >50%

We thank the reviewer for highlighting our omission. 95% confidence intervals have now been included (see Table 13, pages 11 and 12).

6. The paper does not state the level of significance.
   This has now been included in the paper - see page 9, lines 199-200.
7. Uniformly report p-values
   We have reported all p values as their true value, unless it is smaller than 0.001. This is common practice in our field.
8. Report ICC with 95% CI, without F and p-value
   This analysis has now been removed based on the recommendation of Reviewer 1.
9. Add leading zeros to numeric values before of decimal places.

We have now added leading 0s to all decimal places throughout the manuscript.
