# Supplementary material for: The validity and reliability of school-based fundamental movement skills screening to identify children with motor difficulties
Source: PLoS One. 2024 Feb 15;19(2):e0297412. doi: 10.1371/journal.pone.0297412 (PMC10868745; doi:10.1371/journal.pone.0297412)
Supplement: Attachment — Submitted filename: Response to Reviewers.docx [file pone.0297412.s002.docx]

Abstract
 1. The separation between inter-rater reliability (Kappa of researcher vs teachers ratings) and the predictive validity (FUNMOVES vs MABC-2 in the subgroup) is now clear in the main text. In the abstract however, this sentence is confusing: “Four classes (n=91) were scored by both researchers and teachers to evaluate inter-rater reliability against ‘gold standard’ scoring.” Please delete “against gold standard scoring”. Add in a sentence explaining that the MABC-2 is considered the gold standard in the predictive validity assessment.
We agree this could be confusing, given the ‘gold standard’ terminology used for the MABC. We have now removed this and added in a sentence justifying the use of the MABC for concurrent and predictive validity analyses (see page 2, line 26).

 2. Report 95% confidence intervals for all metrics in the abstract.

95% confidence intervals have been included throughout the abstract. See page 2 lines 29-40.

 3. Use proper language when describing the results. If the authors are describing inter-rater agreement they should not call it “accuracy”, which is a term that indicates that the classification was correct. In this paper, the authors are only assessing if the classifications from different raters were in agreement.

We agree that this could be confusing, given the change of terminology from classification rates to accuracy. This has now been modified(see page 2, lines 28-29).

 4. I am confused by this sentence in the abstract “The Specificity and positive predictive value of FUNMOVES was high (1). Sensitivity and negative predictive values were lower for MABC-2 total score (0.57)”. MABC-2 has been defined as the gold standard. Therefore, by definition, the sensitivity and specificity of the gold standard are both 1.00. The authors should report the metrics (sensitivity, specificity, PPV, NPV) for the FUNMOVES tool in identifying subjects classified with or without the condition (as defined by the MABC-2).

The sensitivity, specificity, PPV, NPV for MABC subscales that are directly comparable to FUNMOVES have been clarified (see page 2, lines 36-39).

Introduction

 5. Introduction is much better now. Page 3, line 56. Two periods.

Thank you for spotting this, one has now been removed.

Materials and Methods

 6. The section 2.2.1 as it is, is not useful. Without specifying which test was considered and which parameters were used, those numbers have no meaning. I understand that the sample size calculation may have been conducted in the design phase when the planned statistical analyses were different from the ones conducted. Either provide a complete description of the rationale for the sample size calculation, or remove the text.

As the sample size calculation no longer relates to the analyses in the paper this section has now been removed (see page 5, lines 107-109).

 7. Please modify Table 1 according to the following suggestions:
 o Rename the column “Description” as “Definition”
 o The definitions reported for sensitivity and specificity are incorrect. Please provide formal definitions. The text as it is, is very misleading. For example: 1) sensitivity is not “ability”, it is a probability. 2) sensitivity is not the probability of the test to correctly identify those who have motor difficulties…it’s the probability of being classified as having motor difficulties by the test GIVEN that the child actually has motor difficulties
 o Add formal definition also for PPV and NPV
 o Classification rate is not a metric. The metric described by the authors is called “Accuracy”. The definition of Accuracy should not be formulated as a question. Please add a formal definition.
 o Add a column, called “Operationalization”. In this column, explain how you calculated the quantities for each metric, exactly as you had done for PPV and NPV in the column currently called “Description”.
 o Remove the “(n=22)”. This is not the appropriate table to report sample sizes.
 o Change the title of the table in accordance with the name given to the analyses in the text

Thank you for this helpful comment - we have made the changes listed above, and agree they increase the clarity of the manuscript for those unfamiliar with these analyses.

 Results

 8. Sample sizes are much more clear now. Please make the following edits to Table 2:
 - Rename “inter-rater reliability” with “Subsample used for inter-rater reliability analysis”. Following the same rationale, also rename “concurrent and predictive validity”.
 - In the first column, below “Sex”, there should be two cells. One cell with “Male, n(%)” and one with “Female, n(%)”. This way, you do not have to repeat in the other three columns “Male: n=” and “Female:=”. This is usually how sex is reported in Tables.

This has now been changed - thank you for this helpful comment, it has improved the readability of the table.

 9. Section 3.2 Inter-rater reliability. There cannot be a gold standard of inter-rater reliability by definition. Please avoid the use of this term when referring to the inter-rater reliability assessment, as it is incompatible with the chosen statistical methods.

This has now been removed.

10. Table 3. Provide confidence intervals for the linear weighted Kappa. Please also delete the mention to “p<.001” in the caption, as no p-value is reported in the table.

Apologies for the oversight, p<.001 has been removed from the footnote, and confidence intervals for the linear weighted kappa have now been included (see Table 3, pages 11-12).

11. Table 4 should be a cross-table with the classification of the FUNMOVES and the one of MABC-2 total score.

We are unsure what is meant by this comment. We inferred this might be a suggestion that we should omit the Aiming & Catching and Balance subscale row. However we have decided to keep this row because it has now been clarified in the methods (see response to comment 13).

12. The concurrent and predictive validity has not been defined as a sensitivity analysis in the methods. Please modify the language.

The table name and text above have now been changed to reflect the content (see page 12, lines 221-225).

13. The MABC-2 Aiming and Catching and Balance subscales (FMS) has not been mentioned at all in the methods and it is unclear where it comes from.
This has now been included in the methods section (see page 9 lines 193-195).

 Discussion

 14. When commenting on the results from the predictive validity analysis, please use correct definitions of the metrics (sensitivity, specificity, PPV, NPV). Do not oversimplify their meaning.The claim “the classification analyses were all strong” is unclear and is not scientific language. (1) Classification analysis is not a recognized term to refer to predictive validity analysis. (2) The phrase “all strong” is nondescript and I therefore don’t know what it means.

We thank the reviewer for highlighting the confusing nature of the discussion around sensitivity, specificity, PPV, NPV. We have edited this section to improve readability and understanding (see pages 14&15, lines 250-275).

15. Page 14, line 278. Should say “could impact the generalizability of results”. Please use appropriate terms for scientific concepts.

The language in this sentence has now been clarified (see page 15, line 284).

16. Page 14, line 283 to page 15 line 288. The fact that demographic factors affect the condition (fundamental movement skill ability levels) does not justify the correction by demographic factors for the test. It has been mathematically proven that correction for demographic factors can reduce the accuracy of tests under certain causal scenarios (this has been shown extensively for cognitive screening tests, for example). It is not “essential to formulate a normative database” until it is shown that the correction for demographic factors improves the metric of interest. This has not been demonstrated in this paper and therefore, it is not true that this “current study shows that such a programme of work is well justified”. In the next paragraph of the discussion, the authors continue to refer to the need for a normative database.
 We appreciate the reviewer's opinion. The wording has been changed, rather than removing this section, because positive results (showing that FUNMOVES may be useful as a screening tool for identifying difficulties, in alignment with a widely used clinical tool) in a preliminary study such as those presented in this manuscript (where the limitations of this are made explicit) do justify further exploration, in larger sample sizes. See page 16, lines 291-295).

17. Line 326. “Psychometric” refers to psychological tests.

The language in this sentence has now been clarified (see page 17, line 332).
