# Supplementary material for: The validity and reliability of school-based fundamental movement skills screening to identify children with motor difficulties
Source: PLoS One. 2024 Feb 15;19(2):e0297412. doi: 10.1371/journal.pone.0297412 (PMC10868745; doi:10.1371/journal.pone.0297412)
Supplement: Attachment — Submitted filename: Response to reviewers.docx [file pone.0297412.s003.docx]

**Rebuttal of reviewer comments**

1. Table 1 still contains problematic statements….

o Operationalization for “Sensitivity” is wrong. Sensitivity is not calculated as “What is the probability that FUNMOVES is correctly classifying children that have motor difficulties (scoring <15th percentile on the MABC)”, it should be “What is the probability that FUNMOVES classifies children as having motor difficulties ([explain what’s the threshold used for FUNMOVES]) given that the children have motor difficulties (they score <15th percentile on the MABC)”.

o Same issue applies to Specificity.

o The definition of PPV is factually wrong. PPV is the probability that a child actually has motor difficulties given that the test classifies the child as having motor difficulties. The operationalization should be: PPV is the probability that children have motor difficulties (they score <15th percentile on the MABC) given that they were classified as having motor difficulties by the FUNMOVES ([explain what’s the threshold used for FUNMOVES]).

o Same applies to NPV

o Accuracy is the probability that a child is correctly classified by FUNMOVES. The operationalization should be “What is the probability that FUNMOVES classifies children in the same way as MABC?”

o All the formulas are wrong,since parentheses are not used. For example, sensitivity is obviously not “TP/TP+FN” but “TP/(TP+FN)”.

o The sentence “Analyses revealed that the probability of FUNMOVES correctly classified all children that do not have motor difficulties” is wrong. This is not the definition of specificity.
o “Additionally, FUNMOVES had a positive predictive value of 1 (95%CI =0.68-1) such that the probability that children who score below the 15th percentile also score below this threshold on the MABC-2 total score is high.” This sentence is also wrong. Please fix it.
o The same applies to the discussion of Sensitivity and NPV.
**We amended these definitions and operationalisations in alignment with the reviewer’s comments from last time.** To avoid confusion, we used the exact terminology from their reviews. They are now suggesting that the wording **they recommended** is inappropriate and 'problematic'. Inconsistencies like this raise concerns that this reviewer is being deliberately obstructive. Papers have been published and highly cited within Developmental Psychology with similar definitions (e.g. Atkinson et al. 2022) which guided the ‘operationalisation’ column in this paper. The reviewer is ultimately arguing over semantics. We have changed these to standardised cited definitions to avoid any further confusion, and aligned the operationalisation of these to Atkinson et al. (2022). We have also included parentheses in the formulas.

2. Page 12, lines 228-234. These analyses have not been explained nor justified. They do not seem to be pre-specified analysis and therefore are questionable.

We responded to the expert advice provided by Reviewer 1 in the first round of revisions to remove regressions and correlations from the paper and refocus the analysis around sensitivity, specificity etc (**which were included in the original manuscript**). In order to undertake these analyses, true positives, true negatives, false positives and false negatives need to be calculated beforehand. These are standard metrics that other papers looking to test agreement use regularly (e.g. Atkinson et al., 2022). Furthermore, their criticism of our lack of pre-registration is extremely unreasonable. We can't make retrospective changes to the analysis used here, which were expressly requested by this reviewer and travel back in time to pre-register them! **This is a criticism that has only arisen because we have been responsive to their early criticisms of our analysis and tried to address these, post-hoc.**

3. Half of the discussion seems to focus on the need of a normative dataset for FUNMOVES to adjust for age. This is not justified by the current work, nor does not seem relevant to the current paper.

There are **four lines** included in the discussion (out of 82 total), outlining the need to understand the applicability of this assessment tool beyond Bradford, in a more generalisable sample. Adjusting for age is not mentioned in these four lines as previous papers e.g. Eddy et al., 2021 (published in PLoS One) found that FUNMOVES does not have item response bias for age and thus there is no need to adjust for age. A normative dataset needs to be created which is nationally representative to gain more accurate percentile ranks for children’s ability levels. We fail to understand how the normal scientific process of generating and reporting positive, small-scale results as a precursor to a large-scale study can be criticised as being “unjustified”.

4. Again, remove “gold standard” from table 3. MABC can be considered a gold standard, but the FUNMOVES assessment of the researcher is not a gold standard.

This is a misunderstanding from the reviewer. Gold standard *scoring* relates to the fact that the researchers involved in the development of the tool have the greatest knowledge on implementation of the tool. We have removed this to avoid confusion.

5. FMS acronym is not defined. I suggest to avoid this acronym and spell the terms out.
   We have removed acronyms throughout the manuscript.
6. Page 8, lines 179-180. RStudio runs using R, please, specify which underlying version of R was used for the analyses
   This has now been clarified in the manuscript.
7. In table 3 the Balance linear Kappa does not have 95% CI
   The authors apologise for this oversight. This value has now been included in Table 3.
