# Supplementary material for: The validity and reliability of school-based fundamental movement skills screening to identify children with motor difficulties
Source: PLoS One. 2024 Feb 15;19(2):e0297412. doi: 10.1371/journal.pone.0297412 (PMC10868745; doi:10.1371/journal.pone.0297412)
Supplement: Attachment — Submitted filename: Response to reviewers_NP.docx [file pone.0297412.s004.docx]

**Rebuttal of reviewer comments**

Reviewer 3

1. The overall premise of the study is good and the reader is able to get the point that the author is trying to make.

We thank the reviewer for their kind words.

2. It is very difficult to understand the numbers and the calculations. A flowchart that includes the numbers in different stages would be helpful for organization purposes and to make the paper easier to read. There are different numbers of participants at the beginning 217 and at the end (22). A flowchart that explains the exclusion of the subjects starting from the top to the end would be helpful. The same applies with to the teachers and researchers. Did the same teacher and researcher pair evaluate different students?

A flow chart was deemed excessive as there were no exclusions and no non-consents. It would only illustrate the number of children on the school roll, as all were invited to participate, flowing to a second box showing the number of children who did participate. Teachers were asked to identify children that they believed were struggling from the total sample (concurrent and predictive) – this has been clarified in the manuscript (see page 10, lines 204-210). In order to test whether teachers and researchers scored the same children in the same way (inter-rater reliability) they had to score the same students.

3. The authors present a table with definitions of sensitivity, specificity, PPV, NPV. In addition to the explanation of these terms, they should put the numbers that applies to their study in that table.
   This table is within the methods section to explain the definitions to a lay reader. The results are presented in a separate table. We have included reference to the definitions table in as an NB in the footer, to refer readers back to definitions.

Reviewer 4

4. Abstract: FMS to ne written in full.
   We thank the reviewer for highlighting an omission in defining an acronym. We have been through the abstract (and the rest of the manuscript) and removed all acronyms (except the MABC-2 as this is a well-known acronym for a clinically used tool).
5. Introduction: The authors should state in a sentence the difficulties with using MABC especially in school setting.
   We agree that the specific difficulties of implementing the MABC being more explicit within the manuscript would be beneficial. We have now included a sentence about this in the introduction (see page 4, lines 74-75).
6. Methods: There is need to input how the researchers assessed each teacher's competence in using the tool after an hour training.
   Implementation fidelity was not formally assessed, as extensive work was done on this during the development of the tool. A line about this has now been included in the manuscript (see page 7, lines 163-165).
7. The sampling method (convenient) should be stated as part of the limitation to this study
   We agree this is a useful addition – and ties into discussions around generalisability already present within the discussion. We have made it explicit in the discussion that convenience sampling may also contribute to this (see page 15, lines 289-290).
8. Procedure: Line 148: skills not sills

We thank the reviewer for spotting this error – we have now rectified this within the manuscript (see page 7, line 152)

9. Line 92: The school was and not were.

We thank the reviewer for highlighting this error – we have now changed this within the manuscript (see page 5, line 98)

10. Line 167, 200: The school identified 30 children should be changed to the schoolteachers.
    We thank the reviewer for highlighting this discrepancy – we have now changed this within the manuscript (see page 8, line 173)
11. Line 201: 22 completed FUNMOVES due to school absenteeism-This information is not clear. This study was done in a day and no drop out from parental consent. Authors need to clarify the information about school absenteeism.
    We apologise for the ambiguity in this section – we have changed the sentence to incorporate more clarity (see page 10, lines 211-212).
12. There is need to provide some information on the FUNMOVES such as, has it been used in previous study, was there any study that has don similar study.
    Discussion: This seems to be narrowed. There is need for the authors to compare their findings (results) with previous studies).
    The first study looking at the inter-rater reliability, and concurrent and predictive validity of FUNMOVES. Only one previous published study involves the assessment tool (as it is newly developed, and has not been made readily available until psychometric properties are established). It is therefore difficult to make comparisons to other studies, as there are no other relevant publications. We have included a sentence in the methods referring readers to the first FUNMOVES paper – outlining its development and what it incorporates (see page 6, lines 129-130). We have also clarified in the discussion that this is the first study to look at these aspects of validity and reliability (see page 14, line 254).
